# Metformin improves *Mycobacterium avium* infection by strengthening macrophage antimicrobial functions

**DOI:** 10.3389/fimmu.2024.1463224

**Published:** 2024-12-16

**Authors:** Sindre Dahl Mediaas, Markus Haug, Claire Louet, Sissel Gyrid Freim Wahl, Alexandre Gidon, Trude Helen Flo

**Affiliations:** ^1^ Centre of Molecular Inflammation Research, Department of Molecular and Clinical Medicine, Faculty of Medicine and Health Sciences, Norwegian University of Science and Technology (NTNU), Trondheim, Norway; ^2^ Department of Infection, Clinic of Medicine, St. Olavs Hospital, Trondheim University Hospital, Trondheim, Norway; ^3^ Department of Pathology, Clinic of Laboratory Medicine, St. Olavs Hospital, Trondheim University Hospital, Trondheim, Norway; ^4^ Department of Circulation and Medical Imaging, Faculty of Medicine and Health Sciences, NTNU, Trondheim, Norway

**Keywords:** *Mycobacterium avium*, non-tuberculous, Metformin, host-directed therapy, macrophage, mouse

## Abstract

**Introduction:**

The incidence and prevalence of infections with non-tuberculous mycobacteria such as *Mycobacterium avium* (Mav) are increasing. Prolonged drug regimens, inherent antibiotic resistance, and low cure rates underscore the need for improved treatment, which may be achieved by combining standard chemotherapy with drugs targeting the host immune system. Here, we examined if the diabetes type 2 drug metformin could improve Mav-infection.

**Methods:**

Metformin was administered to C57BL/6 mice infected intranasally with Mav and C57BL/6 mice were infected intranasally with Mav and treated with metformin over 3 weeks. Organ bacterial loads and lung pathology, inflammatory cytokines and immune cell profiles were assessed. For mechanistic insight, macrophages infected with Mav were treated with metformin alone or in combination with inhibitors for mitochondrial ROS or AMPK and assessed for bacterial burden and phagosome maturation.

**Results and discussion:**

Three weeks of metformin treatment significantly reduced the lung mycobacterial burden in mice infected with Mav without major changes in the overall lung pathology or immune cell composition. Metformin treatment had no significant impact on tissue inflammation except for a tendency of increased lung IFNγ and infiltration of Mav-specific IFNγ-secreting T cells. Metformin did, however, boost the antimicrobial capacity of infected macrophages directly by modulating metabolism/activating AMPK, increasing mitochondrial ROS and phagosome maturation, and indirectly by bolstering type I immunity. Taken together, our data show that metformin improved the control of Mav-infection in mice, mainly by strengthening antimicrobial defenses in macrophages, and suggest that metformin has potential as an adjunct treatment of Mav infections.

## Introduction

Non-tuberculous mycobacterial (NTM) infections have emerged as significant public health concerns worldwide due to their increasing incidence, complicated diagnosis, longevity of treatment, and risk for treatment failure (1–5). NTM comprise all mycobacterial species other than those of the *Mycobacterium tuberculosis* (Mtb) complex and *M. leprae*; they are ubiquitous in the environment (water and soil) and infection is believed to mostly occur through inhalation of aerosolized bacilli or by gastrointestinal acquisition. Species within the *Mycobacterium avium* complex (MAC) are the most clinically relevant and infections with *M. avium* (Mav) are the most prevalent (3, 6).

Mav infections encompass a wide range of clinical manifestations, affecting both immunocompromised and immunocompetent individuals (3, 7, 8). The prevalence is significant in populations with underlying respiratory conditions, such as bronchiectasis, chronic obstructive pulmonary disease, and cystic fibrosis. Moreover, immunocompromised individuals, including those living with HIV/AIDS, solid organ transplant recipients, and individuals receiving immunosuppressive therapies, are at increased risk (1, 4). Mav infection predominantly affects the respiratory system, leading to a spectrum of clinical conditions, ranging from asymptomatic colonization to chronic and progressive pulmonary disease (3, 7). However, Mav can also cause extrapulmonary infections, affecting the lymph nodes, skin, soft tissues, and disseminating to other organs in severe cases. Mav primarily resides and replicates within macrophages, subverts intracellular trafficking to inhibit phagosome fusion with lysosomes, resists bactericidal mechanisms, and evades both innate and adaptive immune responses (9–16). The intracellular lifestyle combined with a thick and waxy cell wall, inherent drug resistance, and variable response to standard antimycobacterial drugs, make Mav-infections difficult to cure (3, 7). Prolonged treatment regimens are required, often lasting for months and years, with poor treatment adherence, drug-related adverse effects, and low cure rates. Host-directed therapy (HDT) has gained significant attention as a promising approach that targets the host’s immune response to augment antimicrobial treatments and possibly shorten the effective dose or treatment duration (17, 18). Based on their mode of action, many drugs in clinical use for the treatment of unrelated diseases can potentially modulate mycobacterial infections - both the risk of developing disease and how well the infection is handled (17–19). However, whereas several compounds have shown HDT potential in cell cultures, animal models, and clinical trials for the treatment of TB, similar studies for NTM infections are lacking (17, 19, 20).

Here, we have tested the potential of nine compounds with previously documented effects on cellular or pre-clinical mycobacterial infection, to improve the control of Mav-infection in human macrophages. The most effective compound from our initial screen was metformin, a biguanide diabetes drug that has shown beneficial effects in TB infection models (21–23) and is associated with decreased TB severity, improved clinical outcomes, and reduced recurrence, lung damage and inflammation in TB patients (17, 24–31). We demonstrate that metformin improved mycobacterial control in mice intranasally infected with Mav by strengthening antimicrobial defenses such as mitochondrial ROS and phagosome maturation in Mav-infected macrophages.

## Results

### Metformin treatment restricts the growth of Mav in human primary macrophages

Nine compounds were selected based on their proposed modes of action on macrophage defense mechanisms known to be important for controlling mycobacterial infections (**Supplementary Table S1**) and tested for the ability to control intracellular growth of Mav in primary human macrophages. We have previously shown that Mav establishes a protected compartment (MavC) in macrophages within the first day(s) of infection where it evades killing and replicates without eliciting inflammatory responses (12, 13). To target both the initial and persistent stages of infection, and without interfering with phagocytosis, macrophages were infected with Mav104 at a multiplicity of infection (MOI) of 10 for 10 minutes, and excess bacteria were removed before compounds were added, either immediately or 4 days post infection (therapeutically, after the establishment of MavCs). 7 days later the intracellular mycobacterial loads were quantified from cell lysates (colony forming units, CFU). Only the anti-diabetic drug metformin significantly reduced intracellular Mav loads (average reduction of 43% when added concomitantly with Mav (**Supplementary Figure S1A**) and 47% when added therapeutically (**Supplementary Figure S1B**) and was selected for further evaluation of therapeutic potential.

### Metformin treatment reduces the organ bacterial load of Mav-infected mice

**Figure 1:**
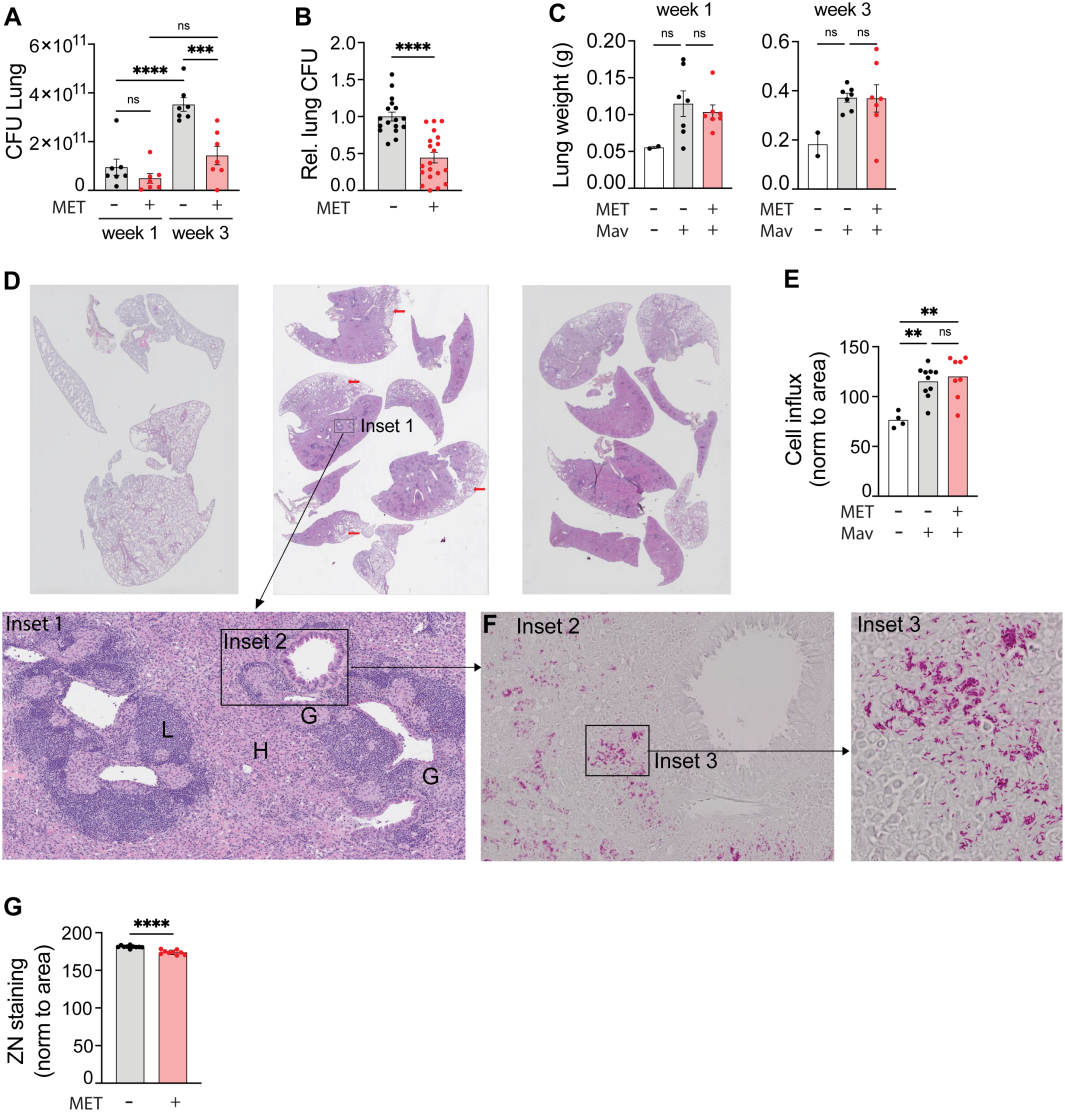
Metformin reduces the lung organ bacterial load of mice infected with *Mycobacterium avium*. C57Bl/6 mice were infected intranasally with 5x10^7^ Mav TMC724 or mock-infected (PBS) and treated 5 times a week with 200 mg/kg metformin (MET) or PBS intraperitoneally over 1-3 weeks. n=7 mice per group in three individual experiments. **(A)** Lung organ bacterial load (CFU) 1 and 3 weeks post infection from one representative experiment. **(B)** Lung CFUs 3 weeks post infection from MET-treated mice normalized to CFUs in mock-treated mice (PBS). Data are combined from three independent experiments. **(C)** Lung weight from the same experiment as in **(A)**. **(D-G)** Organ histology 3 weeks post infection from the same experiment as in **(A)**. **(D)** Hematoxylin and eosin staining of lung tissue sections from uninfected (left) or Mav-infected and mock-treated (middle) or MET-treated (right) mice. Scalebar is xx um. Insets are marked to zoom in on lung structures and bacteria. Inset 1 shows areas of lymphocyte infiltration (L), histiocytes **(H)** and granulomas **(G)**. Scalebar is xx um. **(E)** Morphometric analysis of lung sections shown in **(D)** quantified as cell infiltration relative to the lung area. **(F)** Inset 2 as marked in Inset 1 **(D)**: Ziehl-Neelsen staining of acid-fast bacteria (Mav) in lung granuloma (scalebar is xx um), with magnified Inset 3 (scalebar is xx um). **(G)** Quantification of lung organ bacterial load from Ziehl-Neelsen staining intensity per area in Mav-infected and MET- or mock-treated mice. Significance testing was done using 1-way ANOVA with Tukey’s multiple comparison post-test **(A, C, E)** or students t-test **(B, G)**. **p < 0,01, ***p < 0,005, ****p < 0,001​, ns, not significant. CFU, colony forming units; PBS, phosphate buffered saline; MET, metformin.

A large variety of Mav strains exists in nature that, depending on the virulence characteristics and host susceptibility, can cause disease with varying severity and organ pathology (7). We have previously used a C57BL/6 mouse infection model where intraperitoneal injections of Mav104 result in a systemic, persistent, and non-lethal infection (9, 32). Here, to more closely mimic natural infection, we compared intranasal infection of C57BL/6 mice with Mav104 to the bird isolate Mav TMC724 (MavTMC, equivalent to the ATCC strain 25291), which is shown to be more virulent in mice and causing necrotizing lung granulomas (33, 34). Mice were intranasally infected with Mav104 or MavTMC (1,5x10^7^ (n=3) or 1,5x10^8^ CFUs (n=1)) and the lung bacterial burden was assessed 1 day and 2 weeks post infection (**Supplementary Figure S2A**). Similar lung bacterial loads of Mav104 and MavTMC were retrieved day 1 post infection, but two weeks later the lung burden of MavTMC was increased whereas the opposite was the case for Mav104 (**Supplementary Figure S2A**), suggesting that MavTMC was more efficient than Mav104 in establishing lung infection. Based on these results, MavTMC was selected for further *in vivo* studies of the effect of metformin treatment on Mav infection. Mice were infected intranasally with 5x10^7^ MavTMC and treated 5 times a week with intraperitoneal metformin (200 mg/kg) or PBS as control (mock). After 1- and 3 weeks post infection, animals were sacrificed and the lungs, spleen, and liver were harvested for analysis. Three weeks of metformin treatment significantly reduced the mycobacterial burden in the lung (**Figure 1A**) and spleen (**Supplementary Figure S2B**) compared to untreated mice, with a similar trend toward reduced bacterial loads in the liver (**Supplementary Figure S2B**). We repeated the infection twice for the 3-week timepoint and obtained consistent results with a 40-60% reduction in MavTMC lung burden in metformin-treated mice (**Figure 1B**).

### Metformin treatment does not alter lung pathology or immune cell composition in Mav-infected mice

One of the proposed effects of metformin is reducing inflammation (35, 36), which could be beneficial to prevent tissue pathology but detrimental for mounting an effective host response depending on which inflammatory pathways are affected and the nature/stage of the infection (7, 14, 20, 29, 30). Thus, we next analyzed organ pathology, immune cell infiltration, and inflammation 3 weeks post infection. Mav infection increased lung and spleen weight (**Figure 1C**, **Supplementary Figure S2C**) and lung pathology (**Figure 1D**). Histopathological evaluation of lung tissue sections showed consolidated lung tissue with extensive, confluent infiltrates of histiocytes admixed with mononuclear cells (**Figure 1C** inset 1, *H*), poorly formed granulomas (inset 1, *G*), and nodular lymphocyte infiltrates (inset 1, *L*) in lungs from all infected mice (**Figure 1D**). In some areas there was a sparing of subpleural lung tissue (red arrows). Morphometric analysis of the histological sections confirmed a significant increase in immune cell infiltration per tissue area in lungs from Mav-infected mice compared to non-infected controls, but there was no difference between metformin- and mock-treated mice despite differences in organ bacterial loads (**Figure 1E**). Similarly, image quantification of Ziehl-Neelsen staining (**Figure 1F**, insets 2-3) further confirmed the CFU assessments (**Figures 1A, B**) that metformin treatment reduced the Mav lung burden (**Figure 1G**). Taken together, these results show that metformin significantly reduced the lung bacterial burden without altering the overall immune cell infiltration or lung pathology in Mav-infected mice.

**Figure 2:**
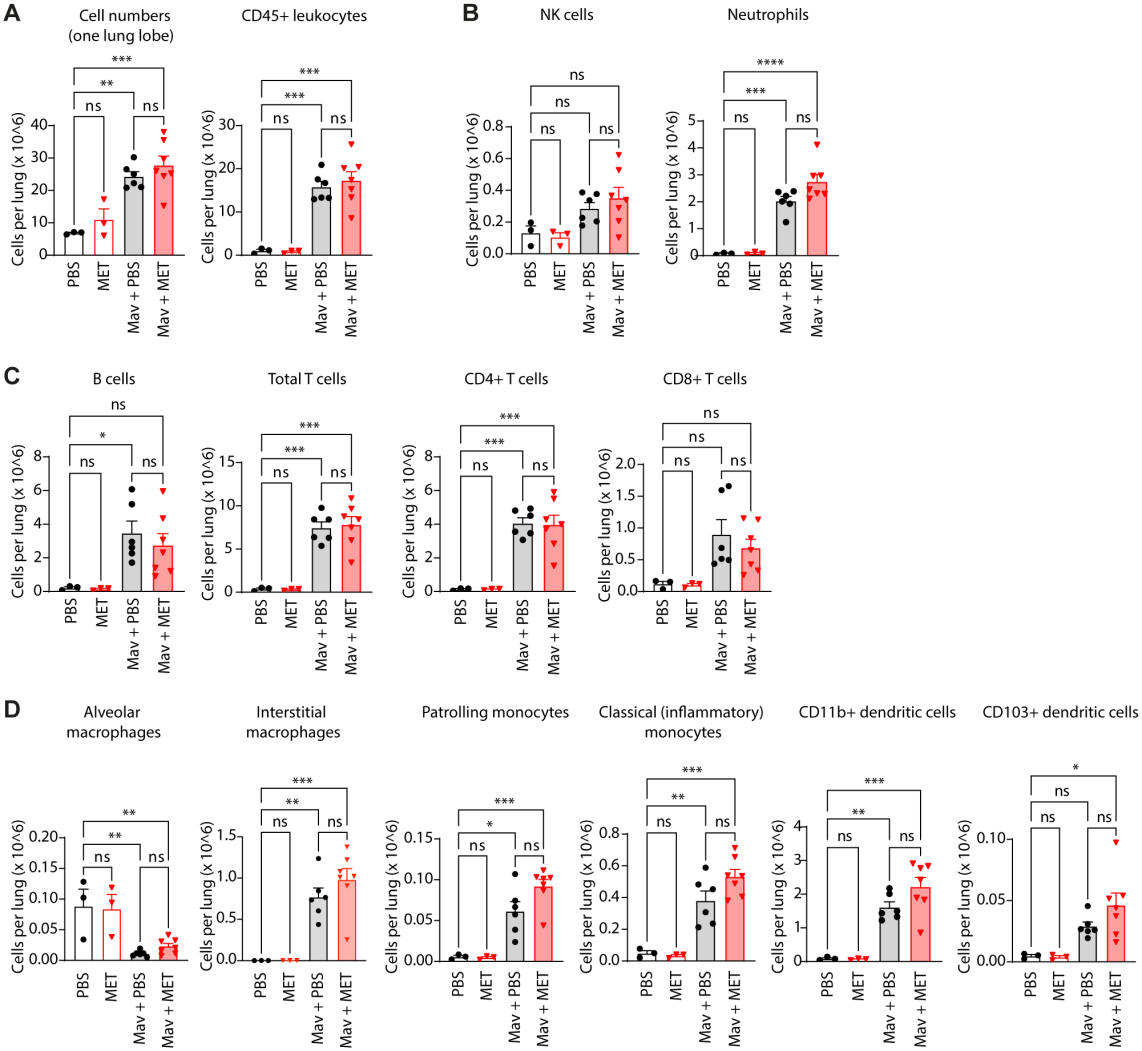
Metformin treatment does not significantly change cell composition in Mav-infected lungs. C57Bl/6 mice were infected with 5x10^7^ Mav TMC724 or mock-infected (PBS) and treated 5 times a week with 200 mg/kg metformin (MET) or PBS (infected: n=7, mock: n=3). Lung cell quantification and phenotyping of lung immune cells were performed 3 weeks post infection. Flow cytometry staining panels are listed in **Table 1** (Methods); gating strategies are shown in **Supplementary Figure S3**. **(A)** Total cell number and total leukocyte numbers per lung. **(B)** NK-cells and neutrophils (staining panel **A**). **(C)** Adaptive immune cell subsets (staining panel **B**). **(D)** Monocyte, macrophage, and myeloid dendritic cell subsets (staining panel **B**). Significance testing was done using 1-way ANOVA with Tukey’s multiple comparison post-test. *p < 0,05, **p < 0,01, ***p < 0,005, ****p < 0,001, ns, not significant.

The lung immune response to mycobacterial infections involves different cells whose contribution can be protective or pathological depending on their relative abundance, activation status, and timing (7, 15, 19, 37, 38). For instance, and on a population level, alveolar macrophages (AMs) are considered anti-inflammatory and permissive for mycobacterial replication whereas recruited, monocyte-derived interstitial macrophages (IM) are generally better capable of restricting infection (39–42), although recent studies have identified both permissive and controller sub-populations of AMs and IMs in TB (42–44). Infiltrating neutrophils engulf mycobacteria and may contribute to early protection, directly or indirectly by interacting with CD4+ T cells, but excessive recruitment of neutrophils correlates with lung pathology in both human and mouse Mav infection (45, 46). Finally, adaptive immunity is generally required to control mycobacterial infections, and in particular Th1-directed cellular immunity with IFNγ-producing CD4+ T cells is thought to be protective whereas the role of other T cell subsets and B cells is still debated (19, 38, 47, 48). Thus, we next evaluated if metformin treatment changed the lung immune cell composition (towards what could be interpreted as a protective phenotype). Total cells isolated from the left lung of each mouse were quantified and stained with two panels of antibodies for identification of the major immune cell populations (methods, panel A) and myeloid cell subsets (methods, panel B) and analyzed using flow cytometry (**Figure 2**, gating strategies in **Supplementary Figure S3**). In accordance with the analyses of lung weight and histology (**Figures 1C, E**), there was a significant increase in total cell number and the number of leukocytes in Mav-infected lungs, but no differences between metformin-treated mice and untreated controls (**Figure 2A**). The same was true for the major immune cell types; NK cells, neutrophils, B cells, CD4+ helper T cells, and CD8+ cytotoxic T cells (**Figures 2B, C**). We further considered the possibility that metformin could modulate the type of immune effector responses and analyzed the Mav-specific T cell effector cytokine production (**Supplementary Figure S4**). As expected (32), there was a substantial increase in IFNγ-secreting CD4+ and CD8+ T cells and of TNFα-secreting CD4+ T cells in Mav-infected mouse lungs, but there was no significant difference with metformin treatment (**Supplementary Figure S4C**). There was, however, a tendency towards an increased percentage of IFNγ-secreting CD8+ T cells in metformin-treated mice compared to untreated controls, as well as a significant increase in the fraction of IL-17-secreting CD4+ T cells only in metformin-treated Mav-infected mice (**Supplementary Figure S4C**). This is in line with the results from Singhal et al. (21) where metformin treatment increased the influx of IFNγ-secreting CD8+ T cells to the lungs of mice infected with Mtb, with a similar trend for IFNγ-secreting CD4+ T cells.

Myeloid cells are particularly relevant since they are the major cell type infected by Mav and can serve as a replicative niche if antimicrobial programs are modulated by the pathogen or not properly activated (9, 10, 12, 15, 49). Metformin treatment did not significantly change the abundance of any of the myeloid cell subsets relative to controls, but there was an overall trend of increased numbers of IMs, patrolling monocytes, classical monocytes, and dendritic cells in lungs from Mav-infected and metformin-treated mice compared to mock-treated controls (**Figure 2D**). Interestingly, and in contrast to the other immune cell populations analyzed, the population of AMs diminished vastly and was almost absent from the lungs of Mav-infected mice three weeks post infection (**Figure 2D**). The results are based on total cell counts and thus cannot be explained by the relative increase in other myeloid cell populations such as IMs, suggesting that the AMs in infected mice either died or downregulated the major AM surface identification marker, Siglec F (**Supplementary Figure S3B, C**). Studies by Bohrer et al. show that host-protective eosinophils are rapidly and substantially recruited to the lungs of Mtb-infected mice, non-human primates, and humans (50, 51). In contrast, at 3 weeks post infection we only observed a small population of CD11b+ Siglec F+ CD11c- CD64- cells/eosinophils that slightly increased upon Mav-infection and was similar in mock- and metformin-treated mice (**Supplementary Figure S3C**).

### Metformin treatment does not alter lung inflammation in Mav-infected mice

**Figure 3:**
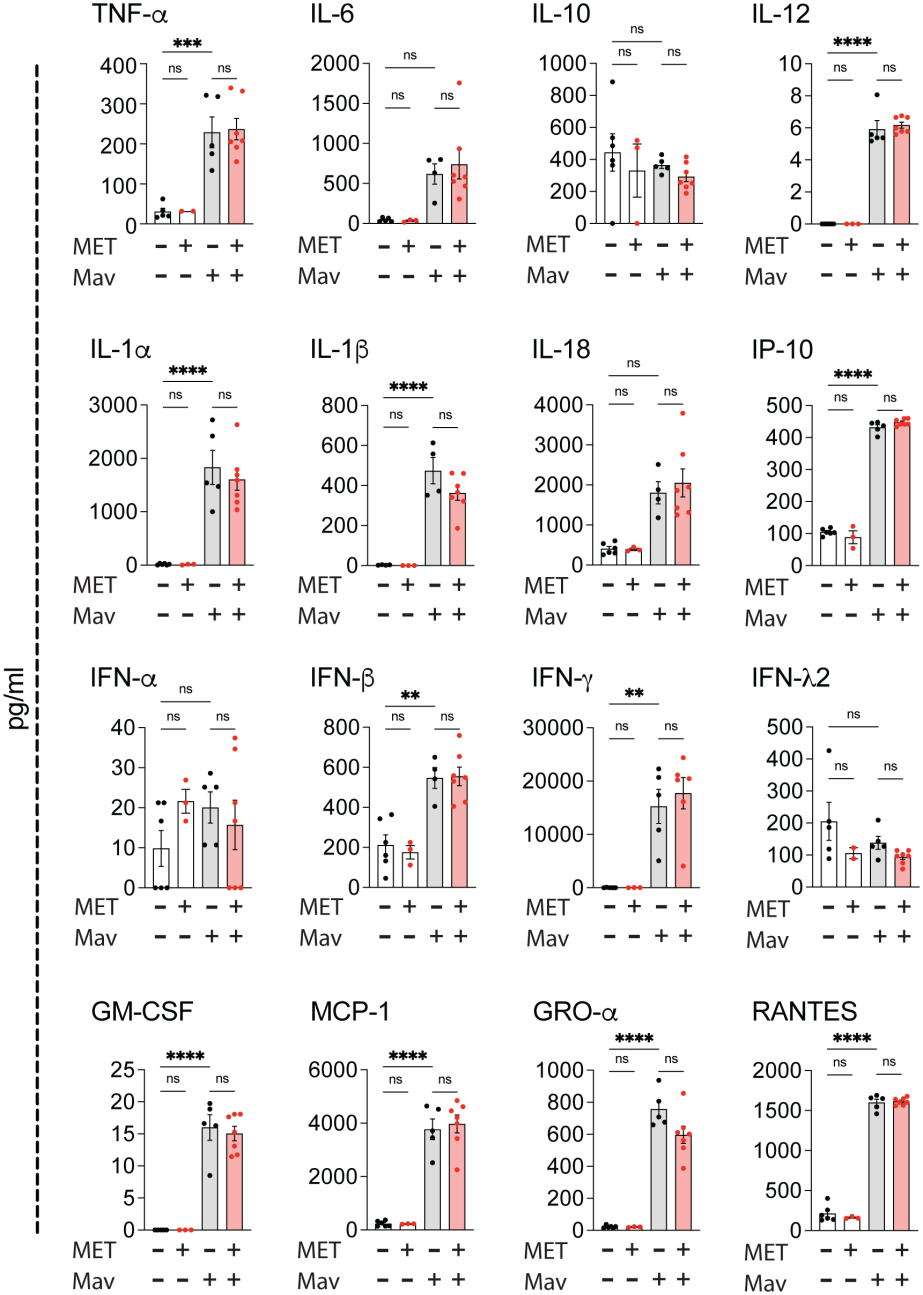
Metformin treatment does not alter cytokine responses in Mav-infected lungs. C57Bl/6 mice were infected with 5x10^7^ Mav TMC724 or mock-infected (PBS) and treated 5 times a week with 200 mg/kg metformin (MET) or PBS over 3 weeks (infected: n=7, mock: n=6 (PBS) or n=3 (MET)). Multiplex cytokine analysis of lung homogenates. Some datapoints are missing due to low bead-counts. Datapoints lower than the detection limit are set to 0. Significance testing was done using 1-way ANOVA with Tukey’s multiple comparison post-test. ** p < 0,01, *** p < 0,005, **** p < 0,001, ns, not significant.

Several inflammatory cytokines are associated with beneficial or unfavorable outcomes in mycobacterial infections depending on the stage of infection and the magnitude/balance of the response mounted: TNFα, IFNγ, IL-12, IL-1, and GM-CSF are generally considered to be protective e.g., by strengthening macrophages’ antimicrobial capacity, directing type 1 immunity, and maintaining granuloma structure/integrity, whereas elevated or sustained type I IFNs can antagonize the responsiveness to IFNγ, modulate eicosanoids and upregulate IL-10 and IL-1Ra, leading to impaired mycobacterial control, increased neutrophil influx and tissue pathology (14, 15, 19, 37, 52). Multiplex analysis revealed substantial cytokine and chemokine levels in lung homogenates 3 weeks post infection, dominated by a strong IFNγ response (**Figure 3**). However, there were no significant differences in cytokine or chemokine levels in metformin-treated mice compared to controls, only a trend of increased IFNγ in metformin-treated compared to mock-treated Mav-infected mice (**Figure 3**). Combined with the tendency of increased Mav-specific IFNγ-secreting T cells in metformin-treated mice (**Supplementary Figure S4**), these results suggest that metformin may strengthen type I immunity in the infected lungs.

### Metformin strengthens macrophage anti-mycobacterial defenses by increasing mitochondrial ROS and phagosome maturation

**Figure 4:**
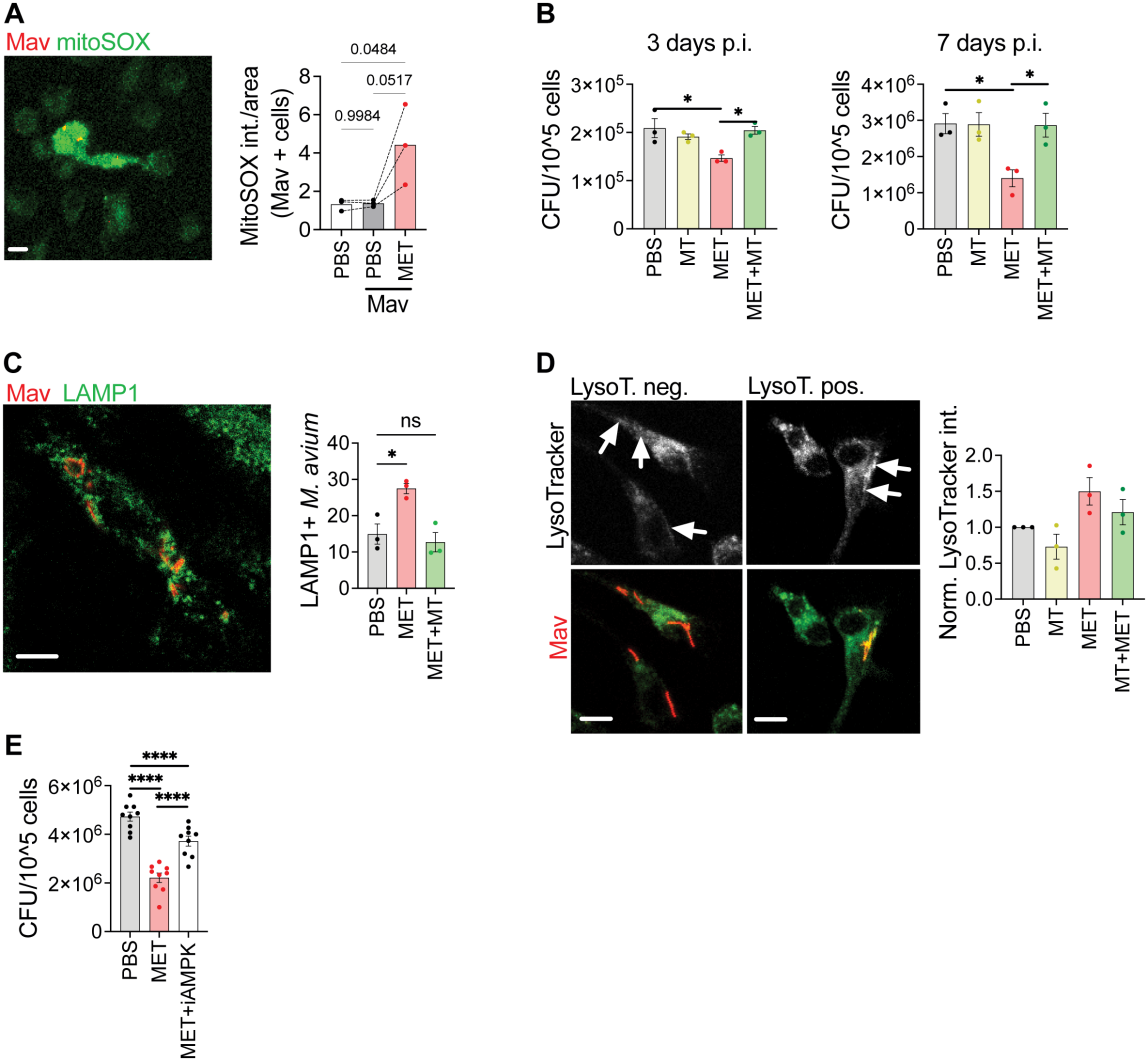
Metformin reduces Mav load in macrophages by increasing mitochondrial ROS, phagosome maturation, and activation of AMPK. **(A)** Mouse BMDMs were infected with Mav104-CFP at MOI 10 for 10 minutes, washed, and treated with metformin (MET, 2 mM) or PBS for 3 days before cells were stained with MitoSOX prior to confocal imaging (n = 3). **(B)** Mouse BMDMs were infected with MavTMC724 at MOI 10 for 10 min and treated with MET (2 mM) and/or MitoTEMPO (10 µM), for 3 (left) or 7 (right) days before cells were lysed and bacterial loads quantified from CFUs (n = 3). **(C, D)** Mouse BMDMs were infected with Mav104-CFP at MOI 10 and treated with MET (2 mM) and/or MitoTEMPO (10 µM) for 3 days before cells were stained with anti-LAMP1 **(C)** or LysoTracker **(D)** prior to confocal imaging (n = 3). **(E)** Mouse BMDMs infected with MavTMC724 at MOI 10 and treated with MET (2 mM) +/- an AMPK inhibitor, Compound C (100 nM) before cells were lysed and bacterial loads quantified from CFUs (n = 9). Significance was tested using 1-way ANOVA with Tukey’s **(A, B, E)**, Dunnett’s **(C)**, or Holm-Sidak’s **(D)** multiple comparison post-test. *p < 0,05, ****p < 0,001, ns, not significant. BMDM, bone-marrow derived macrophage; CFU, colony forming units; PBS, phosphate buffered saline; MET, metformin.

Metformin is shown to strengthen anti-mycobacterial responses such as phagocytosis, reactive oxygen species (ROS), and phagosomal maturation in Mtb-infected macrophages (21, 29, 30, 53), but also to modulate inflammatory cytokines (29, 53). At least some of these effects can be ascribed to metformin inhibiting complex 1 of the mitochondrial respiration chain and activating AMP-activated protein kinase (AMPK), a key regulator of metabolism and mitochondrial homeostasis (21, 29, 30, 53, 54). We have recently demonstrated that mitochondrial ROS (mtROS) contributes to controlling Mav infection in human primary macrophages (55). We next tested if the reduced organ bacterial load in metformin-treated mice could be explained by metformin improving the macrophage control of Mav infection e.g., by increasing mtROS production. Mouse bone marrow-derived macrophages (BMDMs) were infected with Mav104-dsRed (MOI 10) for ten minutes before excess bacteria were removed and cells were treated with metformin for 3 days, stained for mtROS and analyzed by confocal microscopy (**Figure 4A**). Mav infection did not by itself induce mtROS in BMDMs, but metformin increased mtROS in Mav-infected cells (**Figure 4A**). Like in human primary macrophages (**Supplementary Figure S1**), treatment with metformin for 3 or 7 days also significantly reduced the Mav load in BMDMs (MavTMC CFUs in **Figure 4B**, Mav104-CFP fluorescence in **Supplementary Figure S5A**), and concomitant treatment with the mtROS scavenger, MitoTempo (MT), reversed the effect of metformin. MT alone did not change the macrophage mycobacterial load (**Figure 4B**).

Mav needs to be alive to maintain the MavC (12, 13), and if mtROS is mycobactericidal one would expect to see increased phagosomal maturation and acidification in Mav-infected macrophages treated with metformin. This is indeed what we observed: metformin increased phagosome maturation as evidenced by an increased acquisition of the late endosomal/lysosomal marker, LAMP1, to Mav-phagosomes 3 days post infection, and this effect was reversed by MT (**Figure 4C**). Moreover, a trend toward increasing acidification of Mav phagosomes (lysotracker staining) was seen in macrophages treated with metformin compared to untreated controls, which was similarly abolished by MT (**Figure 4D**, **Supplementary Figure S5B**). Metformin is further shown to activate AMPK, both directly (56) or indirectly by targeting the mitochondrial complex 1, which will reduce ATP and increase ADP and AMP (54, 57, 58). We found that a specific inhibitor of AMPK prevented the effect of metformin on reducing Mav intracellular loads, like what we observed with MT (**Figure 4E**, **Supplementary Figure S5B**). Metformin has also been suggested to affect Mtb directly by inhibiting NDH-1, thus affecting the bacterial respiratory chain (59). However, metformin did not impact the growth of Mav in culture, and neither did the AMPK inhibitor (**Supplementary Figures S5CD**). Taken together our results suggest that metformin strengthens the macrophage antimycobacterial capacity by modulating cell metabolism resulting in increased mtROS, phagosomal maturation, and acidification.

### Metformin monotherapy restricts Mav growth but does not significantly enhance the efficacy of conventional antibiotic treatment

**Figure 5:**
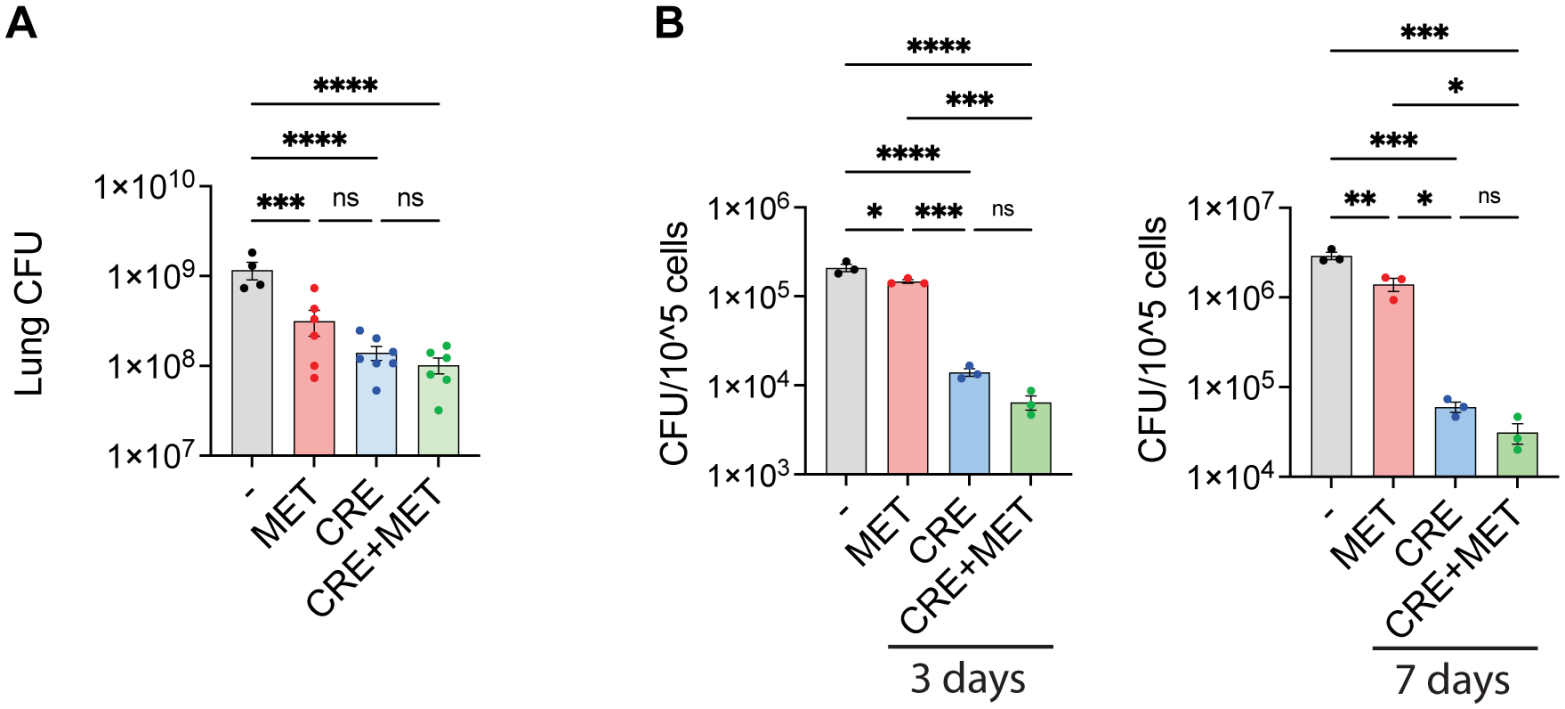
Antibiotic co-treatment with metformin. **(A)** Lung organ bacterial load. C57Bl/6 mice were infected intranasally with 5x10^7^ Mav TMC724 and treated 5 times a week with PBS, 200 mg/kg MET alone or in combination with 200 mg/kg Clarithromycin, 10 mg/kg Rifampicin and 50 mg/kg Ethambutol (CRE) over 3 weeks before organ bacterial loads were assessed from lung homogenate CFUs. n=7 mice per group. **(B)** Mouse BMDMs were infected with MavTMC724 MOI 10 for 10 minutes, washed, and treated for 3 or 7 days with 2 mM MET, 20 µg/ml Clarithromycin, 10 µg/ml Rifampicin and 20 µg/ml Ethambutol (CRE) or CRE+MET prior to cell lysis and CFU plating. Significance was tested using 1-way ANOVA with Tukey’s multiple comparison post-test. * p < 0,05, ** p < 0,01, *** p < 0,005, **** p < 0,001, ns, not significant. BMDM, bone-marrow derived macrophage; CFU, colony forming units; PBS, phosphate buffered saline; MET, metformin; CRE, Clarithromycin, Rifampicin, Ethambutol.

HDTs are not expected to be sufficient as stand-alone treatments but rather as supplements to increase efficacy or reduce the dose or duration of conventional chemotherapy (17, 19, 20). We thus evaluated the *in vivo* and *in vitro* efficacy of metformin treatment in combination with the first-line treatment for Mav pulmonary disease, which is Clarithromycin, Rifampicin, and Ethambutol (CRE) (7). Mice were infected intranasally with Mav and treated intraperitoneally 5 times a week with combinations of metformin and suboptimal concentrations of CRE (25% of the recommended dose). 3 weeks post infection, significant reductions in lung bacterial burden were seen in mice treated with metformin alone, CRE alone, or the combination of the two, but there were no significant differences between treatments (**Figure 5A**). Similar results were obtained *in vitro* from BMDMs 3 or 7 days post Mav infection (**Figure 5B**), although here, CRE was significantly more efficient than metformin in reducing the Mav burden, and a trend of improved CRE efficacy with metformin adjunct treatment was observed.

## Discussion

The increased prevalence of NTM infections combined with insufficient treatment options and poor treatment outcomes support the search for new therapeutic approaches, including those aimed at improving host defenses as an adjunct to conventional antimicrobial treatments (17, 18, 20). HDTs can modulate the inflammatory response to reduce tissue damage (18), target specific pathways to skew the immune response, interfere with host factors required by a pathogen to replicate, and/or augment cellular antimicrobial mechanisms. We found that metformin significantly reduced the lung bacterial burden without altering the overall immune cell infiltration or lung pathology in Mav-infected mice. Mechanistically, metformin strengthened the antimicrobial capacity of infected macrophages directly by increasing mitoROS, and indirectly by boosting type I immunity. Our data thus suggest that metformin shows some promise for host-adjunctive treatment of Mav infections.

Metformin is primarily used to manage hyperglycemia in patients with type 2 diabetes. However, recent studies have unveiled its intriguing potential as an HDT for mycobacterial infections (17, 20, 21, 29, 30, 53). The dose of metformin used in our study (200 mg/kg) is equivalent to 975 mg/day for a 60-kg human (60) and within the recommended daily doses for type 2 diabetic patients (500-2000 mg/day). However, we administered metformin intraperitoneally 5 days per week instead of daily peroral, which could affect the pharmacokinetics and the effective lung tissue concentration during the experiment. Although we found that metformin significantly reduced the lung bacterial burden, the variability in organ CFUs among metformin-treated mice seemed higher than in mock-treated mice. Thus, since metformin has a short half-life (hours), it could be that i.p. administration of metformin 5/7 days per week created variability in our results, and/or the dose used was suboptimal to maintain therapeutic levels.

The precise molecular mechanism behind the protective effect of metformin in Mav infection is difficult to decipher: Metformin is reported to target different cellular pathways, including metabolism, gluconeogenesis, inflammatory cytokine production among others, most likely via different molecular targets depending on the cell and organ (35). The general *in vivo* relevance of these mechanisms is discussed since most of the clinical studies of metformin in TB are of hyperglycemic diabetes patients, and the systemic concentration of metformin in patients may be too low to induce the proposed effects observed *in vitro* (61). In mouse macrophages, metformin increased mtROS, and in line with our previous findings in human primary macrophages (55), mtROS contributed to controlling Mav infection. Metformin also increased phagosomal maturation and acidification, which was dependent on mtROS. Our results are thus in line with Singhal et al. who showed that metformin reduced the growth of Mtb in macrophages through increased production of mitoROS and activation of AMPK (21), but the mechanism(s) for how mitoROS or AMPK activation increased mycobacterial control were not further elucidated. We have previously shown that Mav needs to be alive to maintain the MavC, and antimicrobial treatment e.g., with antibiotics, results in MavC fusion with lysosomes and the killing of Mav (12, 13). A plausible explanation is thus that mtROS directly impairs the viability of Mav, which subsequently increases phagosomal maturation. Inhibition of AMPK also reduced Mav growth inside macrophages, which could be a downstream effect of metformin targeting mitochondrial complex 1 resulting in increased AMP: ATP and ADP: ATP ratios and phosphorylation of AMPK (54, 58). However, metformin has also been shown to activate AMPK directly (56) or via a lysosomal pathway (62) thus we cannot exclude that AMPK is activated via non-canonical mechanisms and contributes to mycobacterial defenses in parallel to increased mitoROS. AMPK is a key regulator of cellular metabolism (54, 58) and as it inhibits mTOR signaling, autophagy is induced which may be protective by degrading intracellular bacteria, damaged organelles, or inflammatory complexes such as inflammasomes (63, 64). However, unlike Mtb, which can rupture phagosomal membranes and get exposed to cytosolic sensors, including the autophagic machinery (65–67), Mav lacks ESX-1/PDIMs and remains phagosomal, and we have never observed Mav or MavCs enclosed in LC3B-positive compartments (13). It is thus unlikely that the anti-mycobacterial effect of AMPK is xenophagy of Mav. In fact, AMPK phosphorylates more than 100 different target proteins, including acetyl-CoA carboxylase and 3-hydroxy-3-methylglutaryl-CoA reductase, which results in the inhibition of fatty acid- and cholesterol synthesis (58) and could thus reduce nutrient availability for intracellular Mav (11, 68, 69).

Metformin-treatment is also reported to modulate inflammation both *in vitro* and *in vivo* (35, 36, 70) including in Mtb-infection (21, 53). In contrast, we did not observe any major impact of metformin on lung inflammation in Mav-infected mice except a tendency of increased IFNγ, including in IFNγ-producing CD8+ T cells. Our results are thus in line with those of Singhal et al. that metformin may strengthen type I immunity in mycobacterial infection (21), and later studies by the same group show that metformin expands a population of antigen-inexperienced memory-like CD8+ T cells with anti-mycobacterial properties (23). However, our results differ in that we did not observe a reduction in lung inflammatory cytokines or tissue pathology in infected mice. The reason for these discrepancies could be the higher dose of metformin used by Singhal et al. (500 mg/kg vs 200 mg/kg in our study) and that Mtb and Mav differ in virulence and inflammatory potential, especially related to the expression of ESX-1 which is lacking in Mav. Thus, we believe the protective effect of metformin in reducing the lung bacterial load of Mav-infected mice is mainly by improving type I immunity and the antimicrobial activity of macrophages. Interestingly, Lachmandas et al. recently showed that peripheral blood mononuclear cells from healthy humans receiving metformin had increased phagocytosis of Mtb, activation of AMPK and ROS production, but reduced OXPHOS and inflammatory signaling including type I IFNs (53). Although not investigated in the present study, it would be interesting to isolate macrophages from the metformin-treated mice to test their inflammatory, phagocytic, and anti-microbial capacity *in vitro*.

We observed an almost complete depletion of AMs from Mav-infected mice starting one week post infection without any replenishment over the three weeks of infection. This was surprising. AM depletion is described in various infectious and inflammatory conditions such as post-influenza infection (mouse models) (42, 71–73), and seems to be different in different mouse strains e.g., Balb/c mice vs. C57BL/6 mice (74), and the type/strength of the inflammatory stimuli. However, replenishment of the AM population normally happens 2-4 weeks post infection, either by self-proliferation of the tissue-resident AMs or by re-differentiation of infiltrating monocytes (72, 73). It would thus be interesting in future studies to investigate if the AM population would re-establish at later time points, and from which cells. In contrast to our results, both AMs and IMs increased progressively over 8 weeks in a recent study by Kajiwara et al. of intratracheal Mav infection in Balb/C mice (75). Several differences could explain the discrepancy to our study, e.g. different Mav strain, mouse strain, and gating strategies for defining macrophage populations: whereas they defined AMs as CD11c-CD11b+ macrophages, we used Siglec F+ as a main criterium for gating AMs (which were in addition CD11c+CD64+ and CD11b-/low, **Supplementary Figure S3**). Huang et al. (40) similarly describe a small decrease in AMs and a profound increase in IMs 2 weeks post Mtb-infection, although the AM depletion was less pronounced than what we observed in Mav-infected mice 3 weeks post infection.

The mechanism behind AM depletion is poorly described and probably differs depending on the causative infection/inflammation and the context: AMs could die from being infected or from exposure to local inflammatory cytokines [such as IFNs (74, 76)] or depletion of survival factors such as GM-CSF (77–79), or they could migrate into the lung tissue and differentiate into a phenotypically different cell type lacking the typical AM surface markers such as Siglec F (42). Cohen et al. showed that during mouse infection with aerosolized Mtb, uninfected AMs remained in the airway and only infected AMs translocated into the interstitium where the infection spread to new cells, primarily neutrophils and recruited monocytes (39). The translocation was dependent on Mtb ESX-1 for inflammasome activation and secretion of IL-1β;, whereafter IL-1R signaling in non-hemopoietic bystander cells facilitated translocation of the Mtb-infected AMs. Mtb dRD1 was found in airway AMs, not interstitial AMs, and the study does not inform whether there was an overall AM depletion in Mtb dRD1-infected mouse lungs. Like Mtb dRD1, Mav does not express ESX-1 and is a poor inducer of membrane damage, inflammasome activation, and IL -1β; secretion (10, 12), and should thus remain in airway AMs rather than in AMs translocated into the lung interstitium. We originally planned to sort/track Mav localization during mouse infection but unfortunately failed to fluorescently label MavTMC (despite previous success with other Mav strains). Instead, we examined total immune cells from digested lungs and would not be able to differentiate airway- from interstitial cells. Accordingly, since we see an almost complete depletion of AMs (airway + interstitial), our study suggests that Mav-infected AMs die or downregulate surface markers such as Siglec F used to positively identify them. However, we find the latter likely: although Pisu et al. describe an AM subset (AM2) that loses Siglec F expression when infected with Mtb, this subset is small and most AMs sustain Siglec F expression (42). Thus, we find it most likely that the depletion observed in our study is caused by AM cell death, although this will have to be investigated in future studies along with mechanistic studies of the causative cell death mechanism.

Several studies have documented an association between type 2 diabetes and TB and where metformin has shown protective effects (17, 24–31). Similar studies are lacking for NTMs, although two recent studies show a slightly increased incidence of NTMs in diabetes patients in Taiwan (80) and an increased risk for NTM disease in type 2 diabetes patients with diabetes-related complications in South Korea (81), respectively. Unfortunately, metformin treatment was not separately assessed in any of the studies. HDT studies of NTMs are further complicated by the fact that NTMs are not reportable in most countries, they are environmentally acquired with no human-to-human transmission (82), except perhaps for *M. abscessus* (83) and *M. kansasii* (84) and mostly cause disease in individuals with underlying lung disease or a compromised immune system, which may affect the host response to mycobacteria in different ways. We found that metformin treatment improved Mav control in mice and in macrophages, and although metformin did not significantly enhance standard Mav-therapy (clarithromycin, rifampicin and ethambutol) in our initial experiments, the accumulating evidence of the beneficial effects of metformin treatment in several infectious disease settings, including post-infection morbidities, combined with the good safety profiles, argue for further studies to fully understand the therapeutic potential of metformin as an HDT for mycobacterial infections. Such studies should address limitations in the present study, such as long-term efficacy and safety of metformin use, and a more in-depth analysis of immunomodulatory effects over time. Metformin is not metabolized in mammals and is excreted unmodified in urine, reducing the likelihood of drug interactions. Metformin use is, however, shown to alter the gut microbiota structure and composition and there is a general concern over the accumulation of metformin excreted from humans in wastewaters, where it can be transformed into products that are harmful to aquatic animals and plants (35).

## Materials and methods

### Primary human monocyte-derived macrophages

Buffy coats from healthy blood donors were provided by the Blood Bank (St. Olav’s hospital, Trondheim) with approval by the Regional Committee for Medical and Health Research Ethics (REC Central, Norway, NO. 2009/2245). Informed consent is routinely asked for by the Blood Bank, and buffy coats are made available for research when consent is obtained. Peripheral blood mononuclear cells (PBMCs) were isolated by density centrifugation using Lymphoprep (Axis-shield). Monocyte-derived macrophages (MDMs) were generated by adherence for 1 h in complete RPMI 1640 (680µM L-glutamine and 10 mM Hepes, GIBCO) supplemented with 5% pooled human serum (The Blood Bank, St. Olav’s hospital) at 37°C and 5% CO_2_. After 3 washing steps with Hanks Balanced Salt Solution (GIBCO), monocytes were cultivated for 6 days with a change of medium at day 3 in RPMI 1640/10% human serum and 10 ng/mL recombinant M-CSF (R&D Systems). At day 6 the medium was replaced with RPMI 1640/10% human serum.

### Primary mouse bone marrow-derived macrophages

Post euthanasia, C57BL/6 hind legs (femur and tibia) were placed in 25 ml ice-cold Hanks Balanced Salt Solution (Sigma), and muscle and connective tissue were dissected before the bones were placed in 96% ethanol (VWR) for 1 minute. Epiphyses were cut, and bone marrow was flushed into a 50 ml tube using HBSS. Cells were pelleted by centrifugation at 430 g for 5 min, resuspended in 1 ml red blood cell lysis buffer (eBioscience), pelleted again, and resuspended in RPMI 1640 (GIBCO)/10% Fetal calf serum (FCS, GIBCO)/20% L929 conditioned medium before seeding in sterile non-coated 10 cm dishes (Corning). After 4 days of differentiation, BMDMs were gently scraped off, spun down, and seeded at the desired concentration in RPMI/10% FCS.

### Mav culture and infection of macrophages

Mav clone 104 expressing CFP and Mav TMC724 (kindly provided by prof. Norbert Reiling, Research Center Borstel, Germany) were cultured in liquid Middlebrook 7H9 medium (Difco/Becton Dickinson) supplemented with 0,5% glycerol, 0,05% Tween 80 and 10% albumin dextrose catalase. Cultures were maintained at log phase growth (optical density between 0,3 and 0,6 measured at 600 nm) in a shaking incubator at 180 rpm and 37°C. On the day of infection, bacteria were washed with PBS, sonicated 3 times, and passed through a Gauge 15 needle to ensure single-cell suspension.

To test the host-directed therapeutic potential of select drugs (**Supplementary Figure S1** and **Supplementary Table S1**), MDMs were infected with Mav104-CFP at MOI 10 for 10 min, washed, and treated with Simvastatin (1µM, Sigma Aldrich), GW9662 (1µM, Sigma Aldrich), Mepenzolate bromide (100nM, Supelco), Zileuton (100µM, Sigma Aldrich), Diclofenac (2µM, Sigma Aldrich), Prostaglandin E2 (2µM, Sigma Aldrich), Metformin (2mM, Sigma Aldrich), Imatinib (10µM, Sigma Aldrich) or 2-Aminopurine (100µM, Sigma Aldrich) for 7 days, starting day 0 (concomitantly) or day 4 (therapeutically) before cells were lysed for assessment of bacterial load.

To assess the mechanism of metformin in macrophages (**Supplementary Figure S5**), BMDMs were infected with Mav104-dsRed at MOI 10 for 10 min, washed and treated with metformin (2 mM) and/or MitoTEMPO (10 µM, Sigma Aldrich) or iAMPK (100 nM, Abcam) over 3 days before LysoTracker staining, cell fixation, and confocal imaging.

To test if metformin had adjunct effect on antibiotic treatment (**Figure 5**), BMDMs were infected with MavTMC724 MOI 10 for 10 minutes, washed, and treated for 3 or 7 days with 2 mM metformin, 20 µg/ml Clarithromycin (Sigma Aldrich), 10 µg/ml Rifampicin (Sigma Aldrich) and 20 µg/ml Ethambutol (Sigma Aldrich, CRE) or CRE+metformin before cell lysis and CFU plating.

For all experiments to ensure correct bacterial doses were used, serial dilutions of bacterial inoculum were plated on Middlebrook 7H10 plates (BD Diagnostics) for quantification of colony-forming units (CFU). At the end of the experiments, the supernatant was collected for cytokine analysis, while cells were lysed using 0,05% Triton X-100 (Sigma) in PBS. Cell lysates were serial diluted using 0,05% Tween 80 (Sigma) in PBS and plated in triplicates on Middlebrook 7H10 plates (BD Diagnostics) for CFU measurements (bacterial load).

### Mouse infection

Mouse infection experiments were conducted using 8 weeks old female C57BL/6 mice purchased through NTNUs Comparative Medicine Core Facility (CoMed). The mice had free access to food and water throughout the experiments. At the day of infection, mice were sedated by intraperitoneal injections of 1 mg/kg Domitor + 10 mg/kg Midazolam intraperitoneal mix (provided by CoMed) before 50 µl of 5*10^7^ MavTMC724 or PBS was installed intranasally, followed by subcutaneous injections of 5 mg/kg atipamezole + 0,1 mg/kg flumazenil antidote for rapid awakening. Metformin (Sigma Aldrich) (200 mg/kg) and/or antibiotics (Clarithromycin 200 mg/kg, Rifampicin 10 mg/kg and Ethambutol 50 mg/kg) were administered intraperitoneally, alone or in combination, 5 times a week in a total of 500 µl PBS. Mice were sacrificed at predetermined time points (7 or 21 days post infection), followed by the harvesting of organs. The postcaval lobe from the right lung was placed in 4% formaldehyde (VWR) for histology, the rest of the right lung was weighed and placed in 1 ml ice-cold 0,05% Tween 80/PBS for homogenization. Lung homogenates were used for assessment of cytokine production and serially diluted in 0,05% Tween 80/PBS and plated on 7H10 plates for CFU quantification (lung bacterial load). The left lung was used for cell isolation and phenotyping. The left liver lobe and the spleen were removed and 5mm punch biopsies were procured for homogenization and CFU plating in 0,05% Tween 80/PBS on 7H10 plates. A total of 3 mouse infection experiments were performed with 4-7 mice per treatment group. All mice were treated humanely according to Regulations on the use of animals in experiments (Forskrift om bruk av dyr i forsøk, FOR-2015-06-18-761) and SOPs provided and approved by CoMed. Mice were attended daily and no animals had to be sacrificed due to health concerns. The study was approved by The Norwegian Food Safety Authority (FOTS 15237).

### Organ histochemistry

During organ harvest, the postcaval lobes were removed and fixed with 4% formaldehyde for Hematoxylin and eosin staining and Ziehl-Neelsen staining. Tissue embedding, slicing, and staining were done by the NTNU Faculty of Medicine and Health Sciences imaging core facility, CMIC. Tissue sample slides were digitalized using Olympus VS200 Slide scanner, and histology images were created using Olympus OlyVia V4.1.

### Lung cell isolation

Mouse lungs were flushed with PBS through the right ventricle and harvested. Lung cells were isolated from the left lung of each mouse using a mouse Lung Dissociation Kit (Miltenyi 130-095-927), MAC SmartStrainer 70µm (Miltenyi 130-098-462), gentleMACS C Tubes (Miltenyi 130-096-334) and gentleMACS Dissociator (Miltenyi 130-093-235). Lung cells were resuspended in PBS and total cell numbers were counted using Z series Coulter cell counter, with 1 drop of Zap-OGLOBIN (Beckman Coulter).

### Lung cell phenotyping

Lung cells were stained with a general panel for leukocytes (Phenotyping Panel A) and a separate panel for myeloid cell subsets (Phenotyping Panel B), **Table 1**. Post-staining flow cytometry was performed using gating strategies as listed in **Table 2** on a BD LSR II flow cytometer (BD Biosciences). Data were analyzed using FlowJo software (FlowJo, LLC, version 10.10.0).

**Table 1:** Phenotyping panels for flow cytometry of lung cells.

Phenotyping Panel A				Phenotyping Panel B			
Marker	Producer	Cat nr	Fluorochrome	Epitope	Producer	Cat nr	Fluorochrome
CD45	BioLegend	103138	BV510	CD45	BioLegend	103138	BV510
CD3	eBioscience	25-0031-82	PE/Cy7	F4/80	BioLegend	123147	BV711
CD4	BioLegend	100451	BV605	CX3CR1	BioLegend	149020	FITC
CD8	eBioscience	56-0081-82	A700	CCR2	BioLegend	150604	A647
Ly-6G	BioLegend	127610	A647	SiglecF	eBioscience	56-1702-80	A700
CD49b	eBioscience	48-5971-82	eFluor450	CD64	BioLegend	139307	PerCP/Cy5.5
NK1.1	BioLegend	108728	PerCP/Cy5.5	CD11b	BioLegend	101257	BV605
CD19	eBioscience	11-1093-82	FITC	CD11c	BioLegend	117336	BV785
F4/80	BioLegend	123147	BV711	CD103	eBioscience	12-1031-82	PE
CD11b	BD Biosciences	557397	PE	Ly-6C	eBioscience	48-5932-82	eFluor450
CD11c	BioLegend	117336	BV785	Ly-6G	eBioscience	25-9668-82	PE/Cy7
Viability	eBioscience	65-0865-18	eFluor780	NK1.1	eBioscience	25-5941-82	PE/Cy7
				CD19	eBioscience	25-0193-82	PE/Cy7
				CD3	eBioscience	25-0031-82	PE/Cy7
				Viability	eBioscience	65-0865-18	eFluor780

**Table 2:** Gating strategies of lung immune cell populations.

Cell type	Abbreviated	Gated cell marker​
Panel A - major immune cells		
**CD3+ T cells​**	​T cells	CD45+ CD3+​
**CD4+ helper T cells​**	​CD4+ T cells	CD45+ CD3+ CD4+ CD8-​
**CD8+ cytotoxic T cells​**	​CD8+ T cells	CD45+ CD3+ CD4- CD8+​
**Natural Killer cells​**	NK​ cells	CD45+ CD3- NK1.1+​
**B Cells​**	B cells	CD45+ CD3- CD19+​
**Polymorphonuclear leukocytes**	Neutrophils	CD45+ CD3- Ly6G+​
Panel B - myeloid subsets		
**Interstitial Macrophages​**	IM​	CD45+ CD3- CD19- Ly-6G- NK1.1- CD11b+ CD11c+ CD64+​
**Alveolar Macrophages​**	AM​	CD45+ CD3- CD19- Ly-6G- NK1.1-CD11b- CD11c+ CD64+ SiglecF+​
**Patrolling monocytes​**	Patrolling Mo​	CD45+ CD3- CD19- Ly-6G- NK1.1- CD11c- Ly6C low F4/80- CX3CR1+ CCR2 low​
**Classical inflammatory monocytes​**	Classical Mo​	CD45+ CD3- CD19- Ly-6G- NK1.1- CD11b+ CD11c- Ly-6C++ F4/80 low CX3CR1+ CCR2+​
**CD11b+ Dendritic Cells (tissue-resident, non-lymphoid)​**	CD11b+ DCs​	CD45+ CD3- CD19- Ly-6G- NK1.1- CD11b+ CD64-​
**CD103+ Dendritic Cells (tissue-resident, non-lymphoid)​**	CD103+ DCs​	CD45+ CD3- CD19- Ly-6G- NK1.1- CD11b- CD64- CD103+​

### T cell stimulation and intracellular cytokine analysis

To assess Mav-specific T cell effector cytokine production, isolated mouse lung cells were seeded at 0.5 x 10^6^ cells/well in 96-well plates in 200 µl complete RPMI 1640 supplemented with 10% FCS. Lung cells were left untreated, exposed to MavTMC724 (MOI 3, 20h) or treated with cell stimulation cocktail (eBioscience™ Cell Stimulation Cocktail (500X), 4h). Protein transport inhibitor was added for the last 4h of incubation (eBioscience™ Protein Transport Inhibitor Cocktail (500X)). Cells were stained using Fixable Viability Dye eFluor780 (eBioscience 65-0865-18). Surface staining of CD4 (BV605, BioLegend 100451), CD8 (A700, eBioscience 56-0081-82) and CD3 (FITC, BioLegend 100204) was performed before cells were fixed (2% paraformaldehyde in PBS, 20 min room temperature) and permeabilized (0.5% saponin in PBS 2% FCS). Permeabilized cells were stained for intracellular IFNγ (PE, BioLegend 505808), TNFα (APC, eBioscience 17-7321-82) and IL-17A (PE/Cy7 eBioscience 25-7177-82) and subsequently analyzed on a BD LSR II flow cytometer (BD Biosciences) and analyzed using the FlowJo software (FlowJo, LLC, version 10.10.0).

### Multiplex cytokine analysis

Lung homogenates were assayed for cytokines/chemokines using a custom-made 16-plex (Procartaplex, ThermoFischer). Measurements in samples with low bead-count alerts are excluded. Datapoints lower than the detection limit is set to 0.

### Immunostaining

BMDMs cultivated on glass-bottomed 96 well plates (IBL) were fixed and permeabilized using a standard protocol as previously described (12). Briefly, cells were fixed in 4% PFA (VWR) for 10 min and then incubated in NH_4_Cl for 10 min to quench PFA-induced auto-fluorescence prior to permeabilization with PBS/0.05% Saponin. Cells were next incubated for at least 90 min in PBS/0.05% Saponin/20% human serum to reduce non-specific binding before staining with 1 μg/ml anti-LAMP1 mouse monoclonal (Santa-Cruz, H4A3) in PBS/0.05% Saponin/1% human serum over night at 4°C. Cells were washed with PBS/0.05% Saponin/1% human serum and incubated with 2 µg/ml Alexa Fluor 555–conjugated goat anti-mouse IgG (Thermo Fisher) for 45 min at room temperature, washed again and stored at 4°C in PBS containing Hoechst (Thermo Scientific) for nuclear staining.

### Confocal imaging

BMDMs cultivated on glass-bottomed 96 well plates were imaged with a Zeiss LSM880 confocal microscope with 40x NA=1.4 objective (Carl Zeiss Micro-imaging Inc.). Emissions were collected using GaAsP hybride detectors. The following acquisition parameters were used: 1024*1024 pixels image size, numerical zoom set to 0.6, frame averaging 1, and 3D acquisition to collect the entire cell with a Z-stack step of 0.25 µm. CFP was excited with a 458 nm Argon laser and emissions were collected through a 470-500 nm window. MitoSOX Red (500 nM) and LysoTracker Red (50 nM) (Thermo Fisher Scientific, M36008; L7528) and Alexa Fluor 555 were excited with a 543 nm HeNe lasers and emissions were collected through a 560-610 nm window. Images were analyzed with Image J (NIH).

### Image analysis

Image J (NIH) were used for image analysis. Cells partially outside of field of view were not considered. For quantification of phagosome maturation and acidification, Z-stacks were projected using the “sum” setting. Cells were traced automatically using Cellpose software, before cell outlines were manually verified and images were imported to Image J for further analysis. MitoSox and LysoTracker intensities were quantified using threshold fluorescence values. Mav localization relative to LAMP1 signal was assessed manually from 3D stacks to quantify the percentage of Mav inside phagolysosomes.

### Statistical analysis

Post normality testing a two-tailed t-test and analysis of variance (ANOVA) were used on normally distributed data (with multiple comparison test noted in figure legend). Significant p-values were set as * p < 0,05, ** p < 0,01, *** p < 0,005, **** p < 0,001. All statistical analyses were performed using GraphPad Prism software (version 10).

## Data Availability

The original contributions presented in the study are included in the article/**Supplementary Material**. Further inquiries can be directed to the corresponding author.
