# Supplementary material for: Metformin improves Mycobacterium avium infection by strengthening macrophage antimicrobial functions
Source: Front Immunol. 2024 Dec 16;15:1463224. doi: 10.3389/fimmu.2024.1463224 (PMC11682992; doi:10.3389/fimmu.2024.1463224)
Supplement: Supplementary file 1 [file DataSheet1.docx]

Supplementary Material

**Figure S1. Effect of select compounds with proposed host-directed therapeutic potential on Mav burden in human primary macrophages.** Human primary monocyte-derived macrophages were infected with Mav104 MOI 10 for 10 min and treated with the indicated compounds for 7 days, starting day 0 (concomitantly) (**A**) or day 4 (therapeutically) (**B**) post infection. CFU counts from 6 (**A**) or 5 (**B**) individual experiments (donors) were normalized to untreated controls and are shown with mean values +/- SEM. Significance was tested using 1-way ANOVA with Dunnett’s multiple comparisons post-test.

**Figure S2. Organ bacterial load of Mav-infected mice.** (**A**) C57Bl/6 mice were intranasally infected with Mav strain 104 or TMC724 at two different doses, 1,5x10^7^ CFU (left, n=3 mice per group) or 1,5x10^8^ CFU (right, n=1 mouse per group). Lung organ bacterial loads compared 1 day and 2 weeks post infection. (**B, C**) related to Figure 1. C57BL/6 mice were infected intranasally with 5x10^7^ Mav TMC724 or mock-infected (PBS) and treated 5 times a week with 200 mg/kg metformin (MET) or PBS intraperitoneally over 1-3 weeks. n=7 mice per group in three individual experiments. (**B**) Spleen and liver organ bacterial loads (CFU) 1 and 3 weeks post infection from one representative experiment. (**C**) Spleen weight from the same experiment as in (B). Significance testing was done using 1-way ANOVA with Tukey’s multiple comparisons post-test * p < 0,05, ** p < 0,01. CFU = colony forming units; PBS = phosphate buffered saline; MET = metformin.


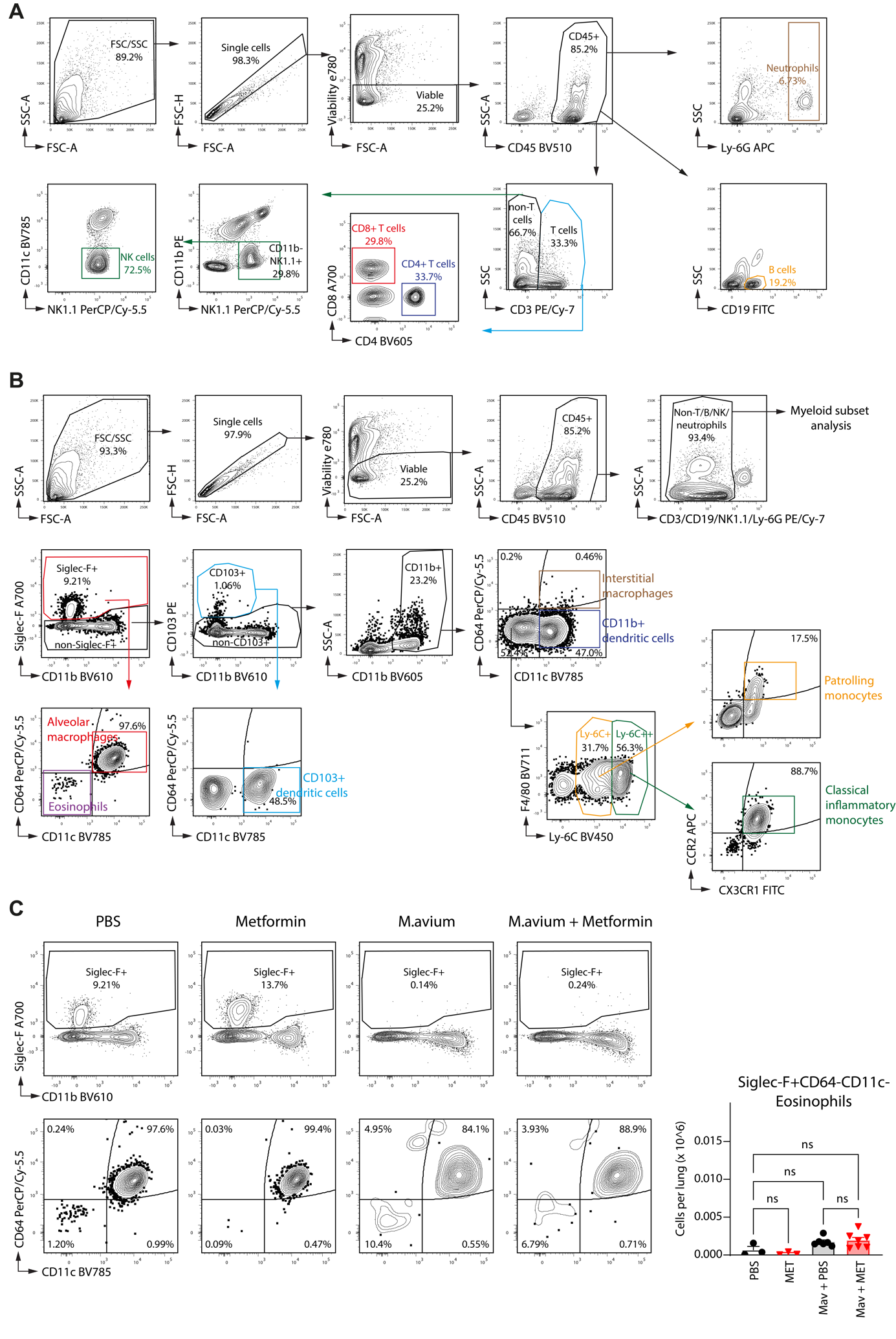


**Figure S3. Lung cell population gating strategies, related to Figure 2.** Gating strategies for phenotyping of lung immune cells by flow cytometry (also see Table 2). Antibody staining panels are listed in Table 1. **(A, B)** Gating for an uninfected, PBS-treated mouse is shown. **(A)** Identification of neutrophils, B cells, NK cells, and CD4+ andCD8+ T cells by staining panel A. **(B)** Identification of macrophage, monocyte, and myeloid dendritic cell subsets by staining panel B. **(C)** Comparison of SiglecF+ lung cells from uninfected and Mav-infected mice with or without metformin treatment 3 weeks post infection. Quantification of SiglecF+CD64-CD11c- eosinophils (infected: n=7, mock: n=3). Significance testing was done using 1-way ANOVA with Tukey’s multiple comparisons post-test.


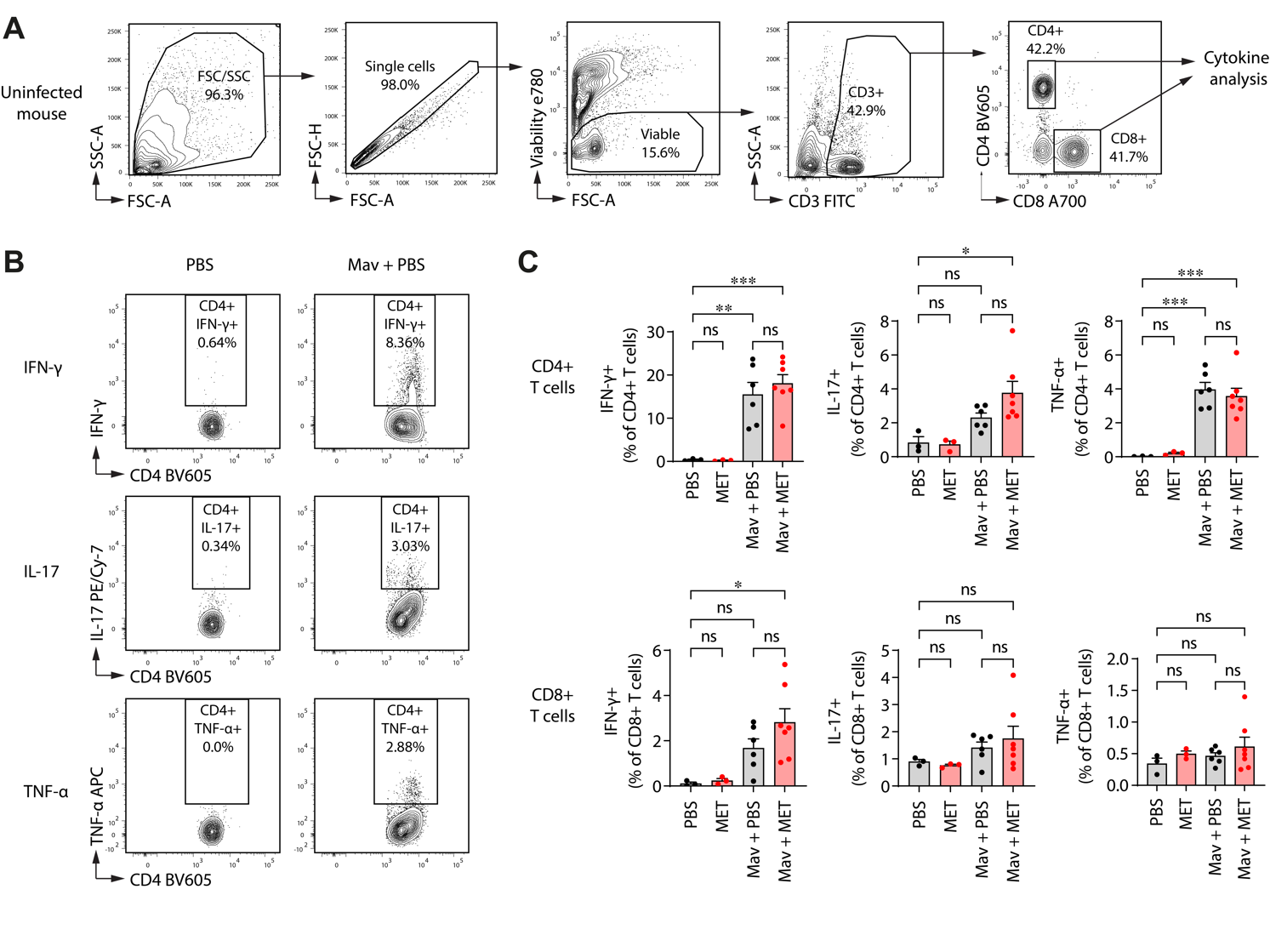


**Figure S4. Metformin treatment does not change effector cytokine responses in lung T cells from Mav-infected mice.** C57Bl/6 mice were infected with 5x10^7^ Mav TMC724 or mock-infected (PBS) and treated 5 times a week with 200 mg/kg metformin (MET) or PBS over 3 weeks (n=7 mice per group). Total lung cells were isolated and stimulated *ex vivo* overnight with Mav before analysis of Mav-specific T cell effector cytokine production by flow cytometry. (**A**) Gating strategy to identify lung T cell populations (uninfected, PBS-treated mouse). (**B**) Example of identification of IFNg-, IL-17- and TNFa-producing CD4+ T cells from lungs of mock-infected (left) or Mav-infected (right) mice. (**C**) Quantification of Mav-specific CD4+ and CD8+ T cells producing effector cytokines IFNg, IL-17 or TNFa. Significance testing was done using 1-way ANOVA with Tukey’s multiple comparisons post-test * p < 0,05, ** p < 0,01, *** p < 0,005.


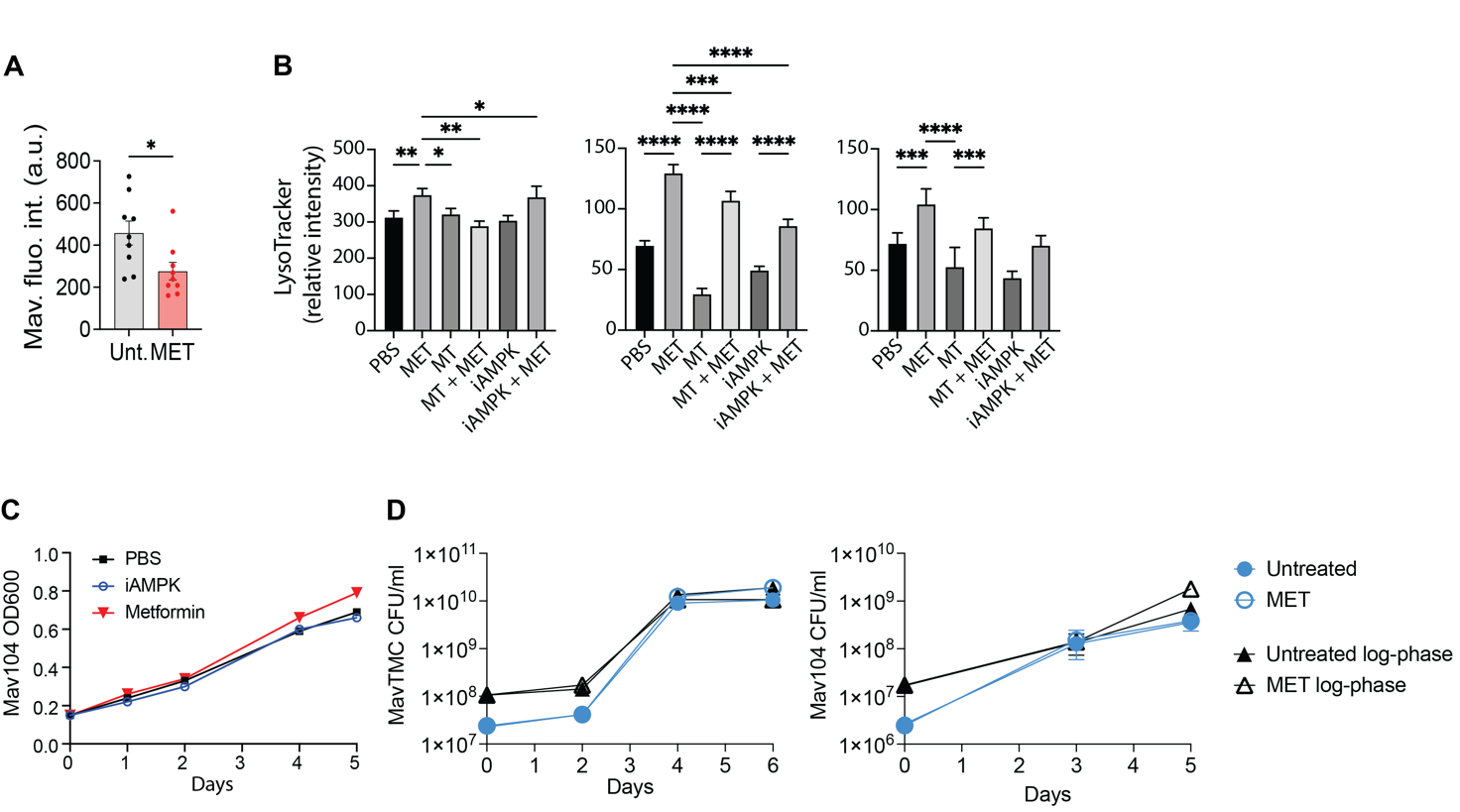


**Figure S5. Inhibitors of mitoROS and AMPK reverse the effect of metformin on Mav load and phagosome acidification in macrophages, related to Figure 4.** (**A**) Mouse BMDMs were infected with Mav104-CFP at MOI 10 and treated with MET (2 mM) for 3 days before cells were stained cells were fixed before confocal imaging measuring Mav fluorescence intensity (bacterial load) (n = 3). (**B**) The effect of metformin on acidification of Mav compartments: individual experiments from Figure 4D. Mouse BMDMs were infected with Mav104-dsRed at MOI 10 for 10 min, washed and treated with MET (2 mM) and/or MitoTEMPO (MT, 10 uM) or iAMPK (100 nM) over 3 days before LysoTracker staining, cell fixation, and confocal imaging. (**C**) MavTMC724 was grown in Middlebrook 7H9 media with the addition of PBS, 2 mM Metformin, or 100 nM AMPK inhibitor Compound C. OD600 measured at the start and daily over 5 days. (**D**) MavTMC724 and Mav104 were grown in Middlebrook 7H9 media, and PBS (closed symbols) or 2 mM Metformin (open symbols) was added to the initial cultures (blue) or to log-phase cultures (black). Samples taken at the indicated time points were plated for CFU counts (n=3 cultures per condition). Significance testing was done using students’ t-test (**A**), Kruskal-Wallis with Dunn’s test for multiple comparisons (**B**), or two-way Anova (**D**). * p < 0,05, ** p < 0,01, *** p < 0,005, **** p < 0,001.

***Table S1*** *Compounds with proposed HDT potential*

| **Compound** | **Mechanism/pathway*** | **Proposed effect on mycobacterial infection*** | **Licensed?** |
| --- | --- | --- | --- |
| **Simvastatin** | HMG-CoA reductase inhibitor | Increased phagolysosome fusion, reduced lipid formation (nutrient availability), immunomodulatory | Lipid lowering |
| **GW9662** | PPARγ antagonist | Increased ROS, inhibition of lipid body formation | No |
| **Mepenzolate bromide** | GPR109A inhibition | Inhibition of lipid body formation, reduced bacterial nutrition | No |
| **Zileuton** | 5-lipoxygenase inhibitor | Modulation of IL-1- PGE2 - type I IFN network (limit excess type I IFN, IL-10, IL-1Ra), promote apoptosis | Asthma |
| **Diclofenac** | Cyclooxygenase 1/2 inhibitor | Anti-inflammatory, reduced synthesis of PGE2, reduced tissue pathology | Pain, fever |
| **Prostaglandin E2 (PGE2)** | Pro-apoptotic eicosanoid | Modulation of IL-1- PGE2 - type I IFN network (limit excess type I IFN, IL-10, IL-1Ra), promote apoptosis | Termination of pregnancy, labor induction |
| **Metformin** | Glucose-lowering | Complex 1 inhibition, AMPK activation, mTOR inhibition, modulate metabolism, anti-inflammatory | Diabetes |
| **Imatinib** | Tyrosine kinase inhibitor | Phagosome acidification, increased myelopoiesis | Leukemia |
| **2-Aminopurine** | Protein kinase R inhibitor | Increased iNOS , TNF and macrophage activation, reduced IL-10, favor apoptosis | No |

* *A description of the HDT potential of the compounds is provided in (1-4)*

**References**

1. Wallis RS, Hafner R. Advancing host-directed therapy for tuberculosis. *Nat Rev Immunol*. (2015) 15:255-63. doi: 10.1038/nri3813

2. Wallis RS, O’Garra A, Sher A, Wack A. Host-directed immunotherapy of viral and bacterial infections: past, present and future. *Nature Reviews Immunology*. (2023) 23:121-33. doi: 10.1038/s41577-022-00734-z

3. Kaufmann SHE, Dorhoi A, Hotchkiss RS, Bartenschlager R. Host-directed therapies for bacterial and viral infections. *Nat Rev Drug Discov*. (2018) 17:35-56. doi: 10.1038/nrd.2017.162

4. Kilinç G, Saris A, Ottenhoff THM, Haks MC. Host-directed therapy to combat mycobacterial infections*. *Immunological Reviews*. (2021) 301:62-83. doi: <https://doi.org/10.1111/imr.12951>
